# Sickness absenteeism in a federal public education institution

**DOI:** 10.47626/1679-4435-2022-796

**Published:** 2023-02-13

**Authors:** Helinton Guedes de Mendonça, Thatiane Lopes Oliveira, Ariane Gonçalves de Oliveira Coutinho, Fabíola Lima Escobar, Karla Jaciara Vieira Damaceno Abreu, Leonardo de Paula Miranda, Pâmela Scarlatt Durães Oliveira, Altamir Fernandes de Oliveira

**Affiliations:** 1 Programa de Pós-Graduação em Educação, Universidade Federal dos Vales do Jequitinhonha e Mucuri, Diamantina, MG, Brazil; 2 Enfermagem, Instituto Federal do Norte de Minas Gerais, Montes Claros, MG, Brazil; 3 Programa de Pós-Graduação em Ciências da Saúde, Universidade Estadual de Montes Claros, Montes Claros, MG, Brazil

**Keywords:** absenteeism, occupational health, surveillance of the workers’, health, absenteísmo, saúde do trabalhador, vigilância em saúde do trabalhador

## Abstract

**Introduction:**

Knowledge on the profile of sickness absenteeism among civil servants reveals their health and working conditions and provides valuable information for the creation of policies aimed at surveillance of servants’ health.

**Objectives:**

To investigate sickness absenteeism in a federal public education institution.

**Methods:**

This was a cross-sectional, documentary, descriptive-exploratory study with a quantitative approach that dealt with the occurrence of sickness absenteeism among federal civil servants at National Institute of Northern Minas Gerais (Instituto Federal do Norte de Minas Gerais).

**Results:**

In the study period, of the total of 1,339 servants, 112 were responsible for 150 episodes of sick leave, which represented a frequency of workers on medical license of 8.36% and a severity index of 3.21 days. Sickness absenteeism was more prevalent among servants aged from 31 to 40 years and among women. Education administrative technicians presented a greater number of leaves when compared to teachers. Mental and behavioral disorders were the most prevalent conditions.

**Conclusions:**

The results of this research may support the creation of more assertive occupational health policies and interventions.

## Abstract (pt)

**Introdução:**

O conhecimento sobre o perfil do absenteísmo-doença entre servidores públicos revela as suas condições de saúde e de trabalho e fornece importantes informações para a criação de políticas voltadas para a vigilância à saúde dos servidores.

**Objetivos:**

Investigar o absenteísmo-doença em uma instituição de ensino pública federal.

**Métodos:**

Tratou-se de um estudo descritivo-exploratório transversal, documental e de abordagem quantitativa, que versa sobre a ocorrência de absenteísmo-doença entre servidores públicos federais do Instituto Federal do Norte de Minas Gerais.

**Resultados:**

No período estudado, do total de 1.339 servidores, 112 servidores foram responsáveis por 150 episódios de afastamentos por motivos de saúde, o que representou uma frequência de trabalhadores com licença médica de 8,36% e um índice de gravidade de 3,21 dias. O absenteísmo-doença esteve mais prevalente entre os servidores com idade entre 31-40 anos e entre as mulheres. Os técnico-administrativos em educação apresentaram um maior número de afastamentos quando comparados aos servidores docentes. Os transtornos mentais e comportamentais foram as condições mais prevalentes.

**Conclusões:**

Os resultados desta pesquisa poderão subsidiar a criação de políticas e intervenções mais assertivas voltadas para a saúde do trabalhador.

## INTRODUCTION

Occupational health represents a range of practices aimed at health promotion, prevention, and surveillance. From this perspective, it is sought to analyze the relationships between workplace and organizational processes, recognizing workers’ reality and determining factors of their health conditions in order to guide decision-making in this area.^1,2^ Therefore, ensuring a safe and healthy environment, which makes workers satisfied with their work activities, may improve productivity and reduce absenteeism rates.^3,4^

Sickness absenteeism is considered a multidimensional phenomenon that evidences occupational disability due to injury or disease and results from the interaction of different labor factors, such as working conditions, but also other aspects inseparable from work, such as psychosocial and economic ones.^3,5^ Recognizing the dialectical nature of the disease process derived from different labor factors, including precarious work, health conditions, and morbidity, leads to a more critical investigation, in order to provide more ground to support occupational health actions.^6^

These actions are important, since the frequent occurrence of sickness absenteeism affects the continuity of work activities by delaying the development of service and overburdening other employees. In public institutions, in addition to affecting productivity and continuity of work, sickness absenteeism causes losses to state coffers due to the costs necessary to rehabilitate servants.^7^

With regard to the theme of this study, there is a scarcity of investigations related to education workers at public institutions, both of the teaching and the administrative staff. However, it can be observed, in the Brazilian public service, that education professionals, together with health care workers, are the main responsible for sickness absenteeism rates.^5,8^

In view of this scenario, studying sickness absenteeism indicators in a federal public education institution, so as to involve all its employees, becomes relevant, since it allows for the creation and strengthening of internal policies aimed at health promotion and surveillance of its workforce. Furthermore, seeking for a situational diagnosis about workers’ health allows for the epidemiological assessment of the group and encourages the establishment of priorities within health promotion and disease prevention programs.^4,8,9^

This study aimed to investigate reasons for sickness absenteeism and its indicators in a federal public education institution. To that end, the following question was raised: how the investigation of sickness absenteeism indicators in a federal public education institution can contribute to actions concerning occupational health?

## METHODS

This was a cross-sectional, documentary, descriptive, exploratory study with a quantitative approach that dealt with the occurrence of sickness absenteeism among federal civil servants at the National Institute of Northern Minas Gerais (Instituto Federal do Norte de Minas Gerais, IFNMG).

The IFNMG consists of a multicurricular higher, basic and vocational institution with a decentralized organization and structured in multiple campuses. Currently, it covers the mesoregions of Northern Minas, Vale do Jequitinhonha, Vale do Mucuri, and also part of Northwestern Minas; moreover, it has two main groups of employees: basic, technical, and technologist education teachers (BTTET) and education administrative technicians (EAT).^10^

The population of this research consisted of permanent servants of the institution with registered sick leaves in 2016, according to the codes assigned by International Statistical Classification of Diseases and Related Health Problems - 10th Revision (ICD-10).^11^ Outsourced employees, who performed work activities at the institution but were linked to other entities, were excluded from the research. The year 2016 was selected because it was the first complete year with the systematic registration of data on sick leaves taken by IFNMG’s employees.

From 05/05/2018 to 05/31/2018, a single researcher performed data collection through documentary search, with access to institutional normative documents. To this end, a database related to servants’ sick leaves was used.

Quantitative data about sick leaves in the IFNMG were obtained from reports generated by the Integrated Human Resource Management System (Sistema Integrado de Administração de Recursos Humanos, SIAPE), as well as by one of its versions, named SIAPE-Saúde, both of them operated by the Federal Government. SIAPE-Saúde is the official federal system for the registration of information on servants’ sick leaves.

In the IFNMG, these sick leaves are evaluated and granted by an official medical expert, and are subsequently included in the aforementioned system. Reports with data on sick leaves were provided by the Coordinating Department of Servant’s Health Care and Quality of Life (Coordenação de Assistência à Saúde e Qualidade de Vida do Servidor, CASQVS), an agency belonging to the organizational structure of IFNMG’s Human Resource Management Directorate Diretoria de Gestão de Pessoas, DGP). These reports were obtained in the pdf format, and data were subsequently transcribed and organized in an electronic spreadsheet by the researcher, for data curation according to study objectives.

In the present study, sick leaves were considered those resulting from health conditions experienced by employees themselves, granted after medical examination and registered on SIAPE-Saúde. Data on employees’ absences related to illnesses of a family member or dependent were not considered in this research.

The collection instrument was designed to meet research objectives and was divided into three items: number of sick leaves (number of granted medical licenses); number of sick-listed servants (number of individuals who took sick leaves); and number of days of sick leave (duration of absence in number of days for sick-listed servants). In addition to the total number of sick leaves, information about age and diagnostic group according to ICD-10 was also investigated.

Furthermore, information on the composition of the institution’s staff, stratified by sex and type of employment relationship, for the calculation of sickness absenteeism indicators of IFNMG’s employees. Indicators were obtained according to the following formulae^8,12^:


Frequency of workers on medical license (FWML)=no. of sick-listed servants×100/total no. of servants in the analyzed period

Absenteeism Severity Index (ASI)=no. days of sick leave/total no. of servants in the analyzed period

To determine the number of working days used to calculate the percentage of absenteeism, weekend days (Saturday and Sunday) were excluded, as well as holidays and official optional working days established for the federal civil servants. Therefore, calculation considered 248 business days in 2016.

Descriptive statistics was used for data analysis. Data were treated using the Microsoft Word and Excel programs, which were used to create spreadsheets and tables in order to present results in percentage and nominal terms, organize them, systematize them, and interpret them on the basis of research objective.

The study project was approved by an Ethics Research Committee (Universidade Federal dos Vales do Jequitinhonha e Mucuri [UFVJM]), duly recognized (opinion no. 2,636,457) by the concerned institution and conducted in accordance with the provisions of Resolution no. 466/2012 of the National Health Council.

## RESULTS

The IFNMG had 1,339 employees in 2016, with a predominance of men in the two categories. There was also a predominance of teachers compared to the number of EAT (Table 1).

**Table 1:** Number of employees at the National Institute of Northern Minas Gerais (Instituto Federal do Norte de Minas Gerais) in 2016

Professional category	Female sex n (%)	Male sex n (%)	Total n
BTTET	274 (38.00)	447 (61.99)	721
EAT	304 (49.19)	314 (50.80)	618
Total	578	761	1.339

Source: Integrated Human Resource Management System (Sistema Integrado de Administração de Recursos Humanos, SIAPE).

BTTET = basic, technician and technologist education teachers; EAT = education administrative technicians.

In 2016, there were 150 episodes of sick leave among 112 IFNMG’s employees, totaling 4,310 days of sick leave, which represents an average duration of sick leave of 28.73 days. Data also show that, of the overall number of sick leaves reported in the institution, 72 (48%) occurred in BTTET, which represented 2,424 days of sick leave. The other episodes of sick leave (52%) occurred in EAT, which totaled 1,886 days of sick leave in this category (Table 2).

**Table 2:** Sick leaves taken by basic, technical and technologist education teachers (BTTET) and education administrative technicians (EAT) in the National Institute of Northern Minas Gerais (Instituto Federal do Norte de Minas Gerais) in 2016, stratified by sex

Category and sex	Number of sick leaves n (%)	Number of sick-list servants n (%)	Number of days of sick leave n (%)
BTTET			
Female	41 (56.94)	28 (52.83)	1.720 (70.95)
Male	31 (43.05)	25 (47.16)	704 (29.04)
Total	72	53	2,424
EAT			
Female	52 (66.66)	39 (66.10)	1,167 (61.87)
Male	26 (33.33)	20 (33.89)	719 (38.12)
Total	78	59	1,886
Overall total	150	112	4,310

Source: Integrated Human Resource Management System (Sistema Integrado de Administração de Recursos Humanos, SIAPE-Saúde).

Women were responsible for most days of sick leave in both categories. Of the total of sick leaves reported in BTTET, 41 (56.94%) occurred in women. With regard to EAT, there was also a predominance of women, with 52 cases (66.66%) (Table 2).

The age group with the greatest number of sick-listed IFNMG’s employees was that from 31 to 40 years (48.10%). This age group was also responsible for the greatest percentage of reported sick leaves (53.33%) (Table 3).

**Table 3:** Percentage of servants and sick leaves in the National Institute of Northern Minas Gerais (Instituto Federal do Norte de Minas Gerais) in 2016, stratified by age

Age	Number of servants	Percentage	Number of sick leaves	Percentage
18 to 30	247	18.45	18	12,00
31 to 40	644	48.10	80	53,33
41 to 50	290	21.66	29	19,33
51 to 59	129	9.63	12	8,00
≥ 60	29	2.16	11	7,33
Total	1.339	100.00	150	100,00

Source: Integrated Human Resource Management System (Sistema Integrado de Administração de Recursos Humanos, SIAPE-Saúde).

The analysis of sick leaves according to CID groups showed that the F00-F99 (Mental and Behavioral Disorders) group was responsible for the greatest number of sick leaves and days of sick leave in the IFNMG in both workers’ categories, accounting for 21.13% of the total of 911 days of sick leave (Table 4). Most ICD groups had a greater number both of sick leaves and days of sickness leave in the female population of servants.

**Table 4:** Sick leaves by groups of the International Statistical Classification of Diseases and Related Health Problems (ICD) in the National Institute of Northern Minas Gerais (Instituto Federal do Norte de Minas Gerais) in 2016, stratified by sex

ICD groups	Number of sick leaves n (%)			Number of days of sick leave n (%)		
	Female	Male	Total	Female	Male	Total
CID F00-F99	25 (26.88)	10 (17.54)	35 (23.33)	663 (22.97)	248 (17.43)	911 (21.13)
CID M00-M99	9 (9.67)	9 (15.78)	18 (12.00)	194 (6.72)	156 (10.96)	350 (8.12)
CID O00-O99	14 (15.05)	-	14 (9.33)	457 (15.83)	-	457 (10.60)
CID S00-T98	8 (8.60)	5 (8.77)	13 (8.66)	316 (10.95)	186 (13.07)	502 (11.64)
CID I00-I99	8 (8.60)	4 (7.01)	12 (8.00)	191 (6.62)	248 (17.43)	439 (10.18)
CID Z00-Z99	8 (8.60)	2 (3.50)	10 (6.66)	444 (15.38)	24 (1.69)	468 (10.85)
CID H00-H59	3 (3.22)	5 (8.77)	8 (5.33)	30 (1.04)	96 (6.75)	126 (2.92)
CID N00-N99	5 (5.37)	3 (5.26)	8 (5.33)	182 (6.30)	66 (4.64)	248 (5.75)
CID H60-H95	3 (3.22)	3 (5.26)	6 (4.00)	81 (2.81)	5 (0.35)	126 (2.92)
CID K00-K93	-	6 (10.52)	6 (4.00)	2 (0.04)-	175 (12.30)	175 (4.06)
CID R00-R99	4 (4.40)	2 (3.50)	6 (4.00)	102 (3.53)	14 (0.98)	116 (2.69)
CID A00-B99	1 (1.07)	4 (7.01)	5 (3.33)	10 (0.35)	58 (4.08)	68 (1.57)
CID J00-J99	1 (1.07)	2 (3.50)	3 (2.00)	5 (0.17)	17 (1.19)	22 (0.1)
CID C00-D48	1 (1.07)	1 (1.75)	2 (1.33)	120 (4.16)	120 (8.43)	240 (5.56)
CID E00-E90	2 (2.15)	-	2 (1.33)	90 (3.12)	-	90 (2.08)
CID G00-G99	-	1 (1.75)	1 (1.33)	-	10 (0.70)	10 (0.23)
CID L00-L99	1 (1.07)	-	1 (1.33)	2 (0.07)	-	2 (0.04)
Other	-	-	-	-	-	-
Total	93	57	150	2,887	1,423	4,310

Source: Integrated Human Resource Management System (Sistema Integrado de Administração de Recursos Humanos, SIAPE-Saúde).

Chart 1 shows the data used to calculate sickness absenteeism indicators in the IFNMG in 2016 and sick leave rates. FWML was 8.36%; and ASI, 3.21 days.

**Chart 1:** Data used to calculate sickness absenteeism indicators and sick leave rates in the National Institute of Northern Minas Gerais (Instituto Federal do Norte de Minas Gerais) in 2016

Total number of servants in the analyzed period	1,339
Number of days of sick leave in the analyzed period	4,310
Number of sick-listed servants in the analyzed period	112
Frequency of workers on medical license	8.36%
Absenteeism severity index	3.21 days

Source: Integrated Human Resource Management System (Sistema Integrado de Administração de Recursos Humanos, SIAPE) and SIAPE-Saúde.

## DISCUSSION

This study allowed for the analysis of important official data from federal servants in two professional categories, producing relevant indicators for the implementation of more assertive occupational health policies and interventions.

During the investigation period, 112 servants were found to be absent for health reasons in the institution, and there were 150 episodes of sick leave. Since the number of sick leaves was higher than that of sick-listed servants, it can be concluded that a single servant took more than one sick leave during the study period. The entire society suffers the impacts of this frequency of absences, because it is not always possible to redistribute the work among the other servants or hiring substitute workers, which affects the quality of the services provided to the population.^13^

With regard to professional categories, although the number of teachers in the institution was higher than that of EAT, the number of sick-listed servants and the number of episodes of sick leave were higher among EAT. However, teachers showed a higher number of days of sick leave, indicating that these professionals spent more time absent from work during the study period. Therefore, this double burden of sickness absenteeism among professional categories eventually compromises both servant’s general health and institution’s holistic efficiency.

EAT work in a variety of positions, having different educational levels and diverse training; however, health-related aspects are still little studied in these professionals. It is known that, due to its multifactorial nature, sickness absenteeism may be related not only to individual worker’s elements, but also to organizational factors.^3,5^ A study conducted with teachers and EAT at a Brazilian public university showed that technicians show a higher frequency of sick leave, occupational accidents, hospitalization, smoking, unhealthy eating, and use of illicit substances, when compared to teachers.^14^

Teacher’s work, in turn, is shown to be different from other work activities, since it requires teachers to have a qualification that goes beyond knowledge belonging to their field of expertise. Several factors may have an influence on teachers’ work routine, impacting on occupational diseases. One of them is the fact that many teachers dedicate most of their time to work, leading to an overload of individual and family demands.^15^ Teachers’ sick leaves occurred recurrently and for the same reasons. This fact suggests that teachers resume their work activities in conditions similar to those that influenced the sick leave.^16^

This vicious cycle favors the onset of somatic and/or occupational diseases, resulting in several harms to teachers and to their performance. Absenteeism is detrimental not only to teachers, but also to the quality of education. The break in continuity of the pedagogical work has a direct impact on students, leading to problems such as low adherence to education and decreased school performance.^16^

It this research, there was a predominance of health-related absences among female servants, despite the higher number of male servants in the study period. The highest incidence of sickness absenteeism among women is a finding corroborated by other studies.^3,5,17,18^ The highest female prevalence seems to be determined by a combination of biological, psychosocial and cultural factors involving the performance of multiple roles.^3,5^ This diversity of factors exposes women to different risk factors. Conversely, women also present higher levels of presenteeism, defined as attending work while sick, which may also intensify the occurrence of a subsequent absence.^19^

With regard to servants’ age, the highest percentage of sickness absence was observed among servants aged from 31 to 40 years, followed by those aged from 41 to 50 years. A similar result was found among public municipal servants^9^; however, in other studies, different results were identified with regard to the predominant age group: 40 to 49 years,^3^ 45 to 51 years,^17^ and 50 to 59 years.^9^ These findings suggest that health-related absences are more frequent among people of more advanced age, partially in contrast with what was observed in this investigation. Furthermore, this research may indicate the existence of a local work context that may be contributing to professional illness among younger individuals.

The literature points out that, as chronological age advances, there is an increase in the frequency of sickness absenteeism, due to worsening of workers’ health status resulting from physiological and social changes in their lives.^5^ It is necessary to investigate the reasons that make servants of not very advanced age to be absent from work due to health reasons at certain institutions, since, in this stage of the life cycle, individuals are expected to have better health conditions. In the case of the IFNMG, the greatest number of episodes of sick leave was observed among workers aged from 31 to 40 years, which may be explained by the presence of a greater number of servants in this age group during the study period.

This research also revealed that mental and behavioral disorders (ICD F00-F99) were the main causes of sick leave, followed by diseases of the musculoskeletal system and connective tissue (ICD M00-M99). This result is consistent with those found in the literature and is supported by other investigations in the field.^5,18,9,20^ Mental disorders are one of the main causes of lost days of work and often become disabling, leading to decreased productivity and evolving to absenteeism.^20^ The results obtained confirm that mental health and work are inseparable in the current productive scenario, including in public services.

Increased work-related stress has stood out as one of the most important causes of mental and behavioral disorders in workers.^20,21^ This stress generates consequences to workers, the organization, and the entire society. When associated with an organic vulnerability, stress may lead to a significant disease or dysfunction in individual’s life. Ideally, professionals should be inserted in an environment with few organizational stressors and be trained to manage and reduce their own stress.^22^

The occurrence of disorders observed in the present investigation is likely to be associated with several stressful factors related to the work routine of civil servants, such as high service demand, accumulation of activities, precarious and deficient physical structures, problems related to public management, political issues that interfere with routines and work processes, among others. Furthermore, other aspects may be involved, such as servants coming from different places, which promotes the feeling of not belonging to the work context; difficulties in adapting to organizational climate; and rupture with the pre-existing social relationships in servant’s place of origin.

All these factors should be considered, since problems related to the workplace, in addition to resulting in reduced work capacity, have a negative impact on the period of medical licenses. Effective intervention in the workplace increases work capacity and improve employees’ quality of life.^23,24^ Hence, there is a strong need for investments in effective policies that act on determinant and conditioning factors of the workplace and also provide mental health support.^20^

With regard to the analysis of absenteeism indicators, 112 servants were absent for health reasons, which represented a FWML of 8.36%. A similar percentage (8%) was found in a study developed by Silva^25^ in a federal higher education institution. However, other investigations showed diverging results. Magalhães^26^ found that sick-listed servants at Universidade Federal do Rio Grande do Norte (UFRN) account for 24% of the total number of servants. In the research conducted by Mesquita,^27^ the number of sick-listed servants at Universidade Federal do Tocantins accounted for 12.87%.

Concerning the number of days of sickness absence in the study period, IFNMG’s employees took a total of 4,310 days of sick leave, thus yielding an ASI of 3.21 days, an index lower than those found in other studies. Magalhães^26^ found an average sick leave duration of 7.08 days per servant at UFRN in 2015. In the study by Bastos et al.,^9^ which aimed to analyze sickness absenteeism in the municipal public service in Vitória, state of Espírito Santo, Brazil, found a mean sickness absence duration of 11.8 days per servant. The treatment of absenteeism is based on the location of several causes internal and external to the service, in addition to individual factors; therefore, workers’ health state is a reason of concern not only to the individual, but also to the organization.^28^

It is worth noting that the data found in this research related to absenteeism indicators in the IFNMG were shown to be lower than those of other studies. One of the factors that may have interfered with values obtained in this research is the possibility of underreporting. A plausible reason for this underreporting is the occurrence of informal agreement often made between servants and their bosses, with the proposal of compensating non-worked days without formal registration of servant’s sick leave, according to legal rules.

Comparison of the findings from this research with those of other studies requires caution, since the characteristics of each institution, as well as the nature of occupations, the work performed, the historical and socioeconomic context, may have an influence on the morbidity profile of organization staff. Any data, regardless of their absolute value, should not be overlooked when dealing with sickness absenteeism at work. Our findings on the theme corroborate the conception that sickness absenteeism has a great impact on the economic and social spheres of organizations and of overall society; additionally, it is necessary to raise awareness on the importance of studying absenteeism in occupational health.^17,18^

Limitations of this study are related to access to restricted registrations on occupational health. The data presented did not allow for establishing direct associations between servants’ health problems and the work they performed. Furthermore, it was not possible to separate occupational diseases from other causes of sickness absenteeism, which would be interesting to investigate the effects of work on the employee’s morbidity profile.

## CONCLUSIONS

Data of this research allowed for the creation of sickness absenteeism indicators in the institution, which may be used to periodically monitor of absenteeism among servants and to support the creation of more assertive policies and interventions aimed at servants’ health promotion and quality of life and at preventing diseases in the working population. It is urgent that the IFNMG, through its departments, managers, and professionals, expand its view on issues involving sickness absenteeism from a perspective that considers not only losses to the organization, but also the human cost resulting from both physical and psychological illness of its employees.

Therefore, information obtained in this investigation may be used as a starting point for other studies, for critical analysis, and for discussion of occupational health public policies that can be replicated nationwide, considering the similarities among federal education institutions in the country. Moreover, our results may encourage studies to adapt international instruments for analyzing absenteeism to the national context. Therefore, the results are expected to help managers formulate preventing measures and develop occupational health programs.
